# The Evaluation of Serum Biomarkers for Non-small Cell Lung Cancer (NSCLC) Diagnosis

**DOI:** 10.3389/fphys.2018.01710

**Published:** 2018-11-29

**Authors:** Rui Fang, Yong Zhu, Vedbar S. Khadka, Fan Zhang, Bin Jiang, Youping Deng

**Affiliations:** ^1^Bioinformatics Core, Department of Complementary and Integrative Medicine, John A. Burns School of Medicine, University of Hawaii, Honolulu, HI, United States; ^2^National Medical Centre of Colorectal Disease, The Third Affiliated Hospital of Nanjing University of Chinese Medicine, Nanjing, China; ^3^Vermont Genetics Network, University of Vermont, Burlington, VT, United States

**Keywords:** lung cancer, NSCLC, serum, biomarkers, diagnosis

## Abstract

**Introduction:** Lung cancer ranks top in the cause of cancer death globally. The identification of effective biomarkers is essential for non-small cell lung cancer (NSCLC) diagnosis.

**Methods:** The expression levels of prolactin (PRL), CEA, and CYFR21 in serum were assayed by ELISA. The blood samples were attained from 44 NSCLC cases and 44 healthy controls. Logistic regression and receiver operating characteristic (ROC) analyses were applied to evaluate the diagnostic efficacy and create diagnostic mathematical models.

**Results:** Serum PRL, CEA, and CYFR21 levels were significantly higher in patients with NSCLC than the healthy controls (all *P*-values <0.001). According to the model to predict NSCLC patients from the healthy controls, a combination of PRL, CEA, and CYFR21 biomarkers was more effective than individual biomarker alone, with AUC = 0.960 (95% CI: 0.921–0.999), sensitivity = 0.909, specificity = 0.955, positive predicted value = 0.952, and negative predicted value = 0.913.

**Conclusion:** Prolactin can be used as a potential serum biomarker for the diagnosis of NSCLC. A panel of PRL, CEA, and CYFRA21 was found as promising serum biomarkers for the diagnosis of NSCLC with relatively high sensitivity and specificity.

## Introduction

Globally, lung cancer is the top cause of cancer death in both men and women, making up 14% of all new cancer and accounting for 1 in 4 cancer deaths (Siegel et al., 2017). Lung cancer is typically comprised of two major categories: non-small cell lung cancer (NSCLC) and small-cell lung cancer (SCLC), which account for about 85 and 15% of lung cancer, respectively. Of NSCLC, two most common subtypes are adenocarcinoma (ADC, ∼70%) and squamous cell lung cancer (SCC, ∼30%). Often, the symptoms of lung cancer do not appear until the cancer has advanced, thus making the early diagnosis difficult. The 5-year survival rate for all people with lung cancer is 18% (Siegel et al., 2017), in part because most patients are diagnosed at a locally advanced or metastatic stage, a point where the curative therapy is no longer available. Currently, the clinical diagnosis of lung cancer mainly relies on chest X-ray, low dose computed tomography (CT) scans and other imaging technology. Unfortunately, the high false positive rates (Shaughnessy, 2017), harmful effect of radiation, and the expense may limit their diagnostic accuracy and utility in widespread lung cancer screening. In addition, there are numerous invasive auxiliary diagnostic methods, such as bronchoscopy and biopsy, but these approaches are painful and laborious. Therefore, the development of non-invasive, sensitive and reliable biomarkers remains a major challenge for researchers.

With the advent of proteomics technologies, a great number of tumor-specific circulating proteins have been recognized from blood samples in recent years (Zamay et al., 2017). Yet, there is no any effective biomarker for the early diagnosis of NSCLC. In NSCLC, serum has been found to be the least invasive and most desirable testing matrix in biomarker evaluations conducted in tissue, plasma, serum, and sputum. Carcinoembryonic antigen (CEA) and cytokeratin 19 fragment (CYFRA21) are among some of serum/plasma lung cancer protein biomarkers that have been most commonly investigated (Crosbie et al., 2013). CEA is a cell adhesion glycoprotein expressed in gastrointestinal tissues at very low levels in healthy individuals (Hammarstrom, 1999). The serum CEA levels were observed significantly higher in NSCLC patients with worse prognosis and poorer survival rates (Cedres et al., 2011). CYFRA21 is a 36 kDa fragment of cytokeratin expressed in epithelial cells. It has been reported as a biomarker of NSCLC that has an independent prognostic role with 59% sensitivity and 94% specificity along with its concentration level reflecting the disease extent (Wieskopf et al., 1995). Combining CEA with CYFRA 21 increased the sensitivity and specificity for the diagnosis of primary lung cancer (Chantapet et al., 2000; Okamura et al., 2013). However, such combination is still unacceptable for the diagnosis of lung cancer due to low sensitivity and specificity. In addition to CEA and CYFRA21, we sought to discover more new and stable serum-based biomarkers such as prolactin (PRL) in breast cancer (Faupel-Badger et al., 2010). In human, PRL is a peptide hormone secreted by the anterior pituitary gland and is known to be expressed in various tissues such as breast and prostate. The associations of PRL and development of various cancer has been evaluated in breast, prostate, colorectal, gynecological, and hepatocellular cancer (Goffin, 2017). Only a few studies have examined the relationship between the PRL levels and lung cancer (Caponnetto et al., 2017; Seder et al., 2017). PRL was introduced in a seven-analyte panel of lung cancer biomarkers for the first time and the panel was suggested useful to risk stratify cancer patients for early recurrence after resection of node-negative NSCLC less than 4 cm (Seder et al., 2017).

In this study, we measured the levels of CEA, CYFRA21, and PRL in a cohort of NSCLCs and healthy controls to investigate the diagnostic efficiency of these markers and to create statistical models to advance in lung cancer diagnosis.

## Materials and Methods

### Patients, Serum Sample Collection, and Preparation

The study was reviewed and approved by Shenzhen Bao’an Shajing People’s Hospital IRB Committee. Patients with clinically ascertained and biopsy-proven untreated primary lung cancer were enrolled from Shenzhen Bao’an Shajing People’s Hospital, China. Both informed and written consents were obtained from all participants. NSCLC was defined based on CT scans and verified by histopathology according to the World Health Organization Classification of Tumors (Travis et al., 2015). Blood samples were collected within 4 weeks from the first biopsy-proven lung cancer diagnosis and prior to removal of cancer by a surgical procedure. Patients had no anti-neoplastic therapy, radiotherapy or chemotherapy before the surgery or diagnosis of lung cancer. A total of 44 healthy blood donors who are on a regular visit to Shenzhen Bao’an Shajing People’s Hospital and with age and sex matching to NSCLC patients were enrolled as healthy controls. The healthy controls had no prior evidence of lung cancer and had not received a diagnosis of malignant or benign tumors including chest X-ray before the blood sample collection. Preoperative peripheral blood samples were collected in anticoagulant-free tubes and treated according to standard protocols. The samples were centrifuged at 3000 rpm at room temperature, divided into aliquots, flash frozen, and stored at -80°C. The clinical characteristics of the NSCLC patients and healthy controls that include sex, age at diagnosis, tumor stage and subtype are listed in Table 1.

**Table 1:** Clinical characteristics of the patients with NSCLC and healthy controls.

	NSCLCs	Healthy controls
No. of patients	44	44
Age in years, mean (SD)	59.8 (9.3)	59.9 (9.6)
Sex, male, n (%)	31 (70.5)	31 (70.5)
Cancer stage, n (%)		
I	13 (29.5)	
II	4 (9.1)	
III	8 (18.2)	
IV	19 (43.2)	
Cancer subtype, n (%)		
Adenocarcinoma	21 (47.3)	
Squamous-cell carcinoma	23 (52.7)	


### ELISA

ELISA kits for PRL, CEA, and CYFRA21 were purchased from Wuhan Yousheng (USCN) Technology Co., Ltd., and applied according to manufacturer protocol. The serum samples and kit components were allowed to equilibrate to room temperature prior to running the assay. Serum aliquots (100 μL) in standard diluent were added to the appropriate well of a 96-well plate, covered with a plate sealer, and the plate was incubated at 37°C for 2 h. The liquid was removed and an aliquot of solution A was added to each well. The samples were again incubated at 37°C for an hour. The plate was washed five times with buffer, and solution B (100 μL) containing hydrogen peroxide at 1:200 dilution was added, and the samples were incubated for 30 min. Colored development was achieved by adding 90 μL 3,3,5,5-tetramethylbenzidine substrate to each well and 50 μL sulfuric acid was added to stop the further reaction. Finally, the optical density at 450 nm was measured in a synergy 2 multimode plated reader.

### Statistical Analysis

The data were summarized by mean with standard deviation (SD) or median with interquartile range (IQR) for continuous variables, and frequency with percentage for the categorical variables. The difference in the serum levels between cancer patients and healthy controls were examined by two-sample *t*-test/Mann–Whitney *U*-test. The association between the outcome variable, cancer or control, and the biomarker levels were then evaluated by the logistic regression. Receiver Operating Characteristic (ROC) curves were plotted. The performance parameters such as sensitivity, specificity, positive predictive value (PPV) and negative predictive value (NPV) were summarized, and area under the ROC curve (AUC) with 95% confidence interval (CI) was calculated to assess the discrimination power of individual biomarker and the combination of biomarkers. All analyses were performed by SAS 9.4 and *P*-value <0.05 was considered as statistical significance.

## Results

### Patients’ Characteristics

The characteristics of the study subjects are summarized in Table 1. The NSCLC patients and healthy controls were comparable in terms of age and sex (both *P*-values >0.05). There were more NSCLC patients in the late stage (43.2%). The distribution of adenocarcinoma (ADC) and squamous-cell carcinoma (SCC) were almost even.

### PRL, CEA, and CYFRA21 in NSCLC Patients and Healthy Controls

**FIGURE 1:**
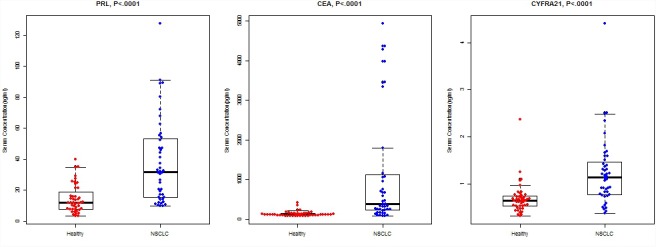
Concentrations of PRL, CEA, and CYFRA21 between NSCLC cases and healthy controls. The bold horizontal lines in the box plots are medians, and the lower and upper limits of the boxes are 25th and 75th percentiles of values, respectively. The *P*-values were obtained from Mann–Whitney *U*-test.

The median serum PRL levels of NSCLC patients were 32.0 (IQR: 15.3–53.3) ng/ml, significantly higher than that of healthy controls (median: 11.9 ng/ml, IQR: 7.9–11.9, *P* < 0.0001, Figure 1). Similarly, the patients with NSCLC had higher serum levels in CEA (*P* < 0.0001) and CYFRA21 (*P* < 0.0001).

### ROC Analyses of PRL, CEA, and CYFRA21, and the Building of Diagnostics Models for NSCLCs

**FIGURE 2:**
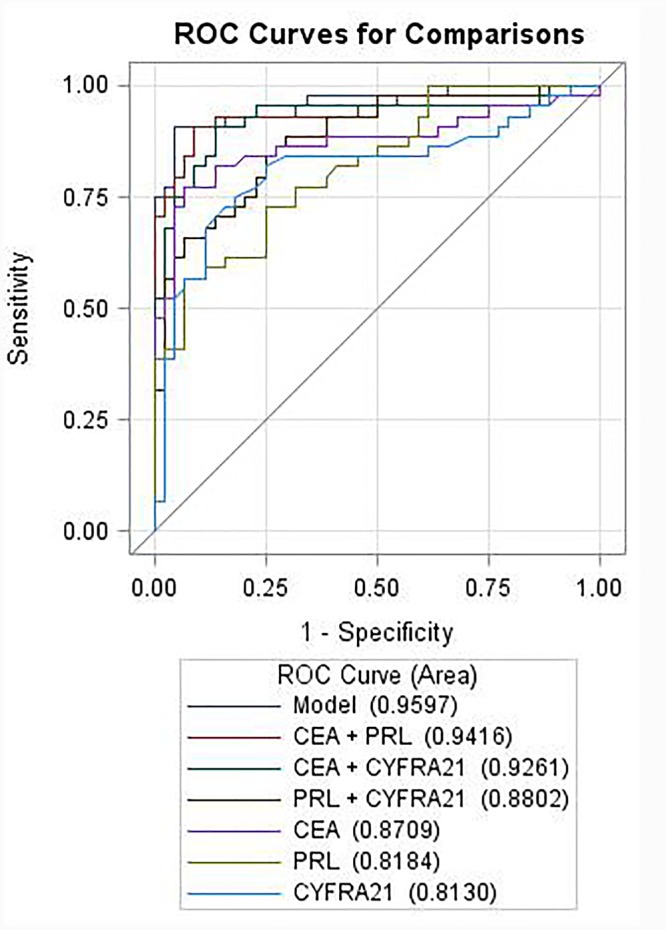
Receiver operating characteristic curves of individual or combination of PRL, CEA, and CYFRA21 serum tumor biomarkers in differentiating NSCLC from the healthy controls.

Among the three serum biomarkers, CEA displayed the highest AUC (0.871, 95% CI: 0.789–0.953) on the training set, followed by PRL (AUC = 0.818, 95% CI: 0.733–0.904), and CYFRA21 (AUC = 0.813, 95% CI: 0.717–0.909). A logistic regression was used to explore whether combining two or three serum biomarkers would improve the diagnostic accuracy. The combination of PRL, CEA, and CYFRA21 yielded a better optimal diagnostic efficacy for cancer patients (AUC = 0.960, 95% CI: 0.921–0.999, Figure 2) than the individual biomarker alone. The ROC curves from ELISA results were plotted to evaluate the diagnostic efficiency. The measurements of the different individual markers and their predictive values in the diagnosis of NSCLCs are summarized in Table 2.

**Table 2:** Diagnostic efficiency of models in differentiating NSCLC from the healthy controls.

	AUC (95% CI)	Sensitivity	Specificity	PPV	NPV
**NSCLC vs. Healthy**					
PRL	0.818 (0.733, 0.904)	0.727	0.750	0.744	0.733
CEA	0.871 (0.789, 0.953)	0.772	0.932	0.919	0.804
CYFRA21	0.813 (0.717, 0.909)	0.750	0.819	0.805	0.766
PRL+CEA	0.942 (0.889, 0.994)	0.909	0.909	0.909	0.909
PRL+CYFRA21	0.880 (0.810, 0.950)	0.863	0.727	0.760	0.842
CEA+CYFRA21	0.926 (0.865, 0.987)	0.909	0.864	0.870	0.905
PRL+CEA+CYFRA21	0.960 (0.921, 0.999)	0.909	0.955	0.952	0.913


*PPV, positive predictive value; NPV, negative predictive value.*

## Discussion

Blood samples, instead of tissue samples from biopsy, are more convenient and non-invasive to collect for testing biomarkers. We sought to evaluate the biomarkers in serum and create a statistical model for discriminating NSCLCs from the healthy controls. Our results confirmed that both CEA and CYFRA21 were raised in patients with NSCLC and potentially be useful biomarkers in serum for the diagnosis of NSCLC. We also observed the serum PRL level was elevated significantly in NSCLC patients with respect to healthy controls, indicating the potential clinical diagnostic relevance of PRL as a serum biomarker of NSCLC patients. Furthermore, the two known clinical biomarkers, CEA and CYFRA21 including PRL were tested in all samples to compare their diagnostic efficacy in differentiating NSCLC from the healthy controls. We determined the AUCs for individual biomarker and their combinations, and selected the appropriate balance between sensitivity and specificity for the cut-off point. These three serum biomarkers when combined had the highest diagnostic value of AUC 0.96 with sensitivity 0.909, specificity 0.955, positive predicted value 0.952 and negative predicted value 0.913 than the individual biomarker alone for NSCLC prediction (Table 2).

Although previous studies suggest that individual serum biomarkers and/or their combinations can distinguish cancer patients from healthy individuals, only a few are in clinical practice. The major constraint on their application is due to lack of sufficient sensitivity or specificity. Several studies have reported results on the evaluation of serum protein biomarker panels associated with NSCLC including CEA and/or CYFRA21. Patz et al. (2007) published a panel of 4 serum proteins comprising retinol binding protein (RBP), 1-antitrypsin (ATT) and squamous cell carcinoma antigen (SCCA) together with CEA which correctly distinguished lung cancer patients on a training set with 89.3% sensitivity and 84.7% specificity; however, there were a slight decrease in sensitivity and specificity on an independent validation set with 77.8 and 75.4%, respectively. Cho (2007) and Cho et al. (2010) reviewed CYFRA 21-1, CEA, SCCA, tissue polypeptide antigen (TPA), cancer antigen 125 (CA-125) as potentially useful biomarkers of NSCLC and later identified elevated human serum amyloid A (SAA) in serum is associated with poor prognosis in lung cancer. Iwahori et al. (2012) found elevated serum human epididymis protein 4 (HE4) levels for NSCLC patients and reported AUC of 0.988 for discriminating lung cancer patients from healthy controls. Nolen et al. (2011) identified a panel with three serum biomarker comprised of MIF, prolactin, and thrombospondin that have high diagnostic utility in lung cancer. The panel was effective in differentiating CT-screened control individuals with suspicious pulmonary nodules and stage 1 lung cancer patients with 74% sensitivity, 90% specificity and 86% accuracy on the training set and 70% sensitivity, 93% specificity and 82% accuracy on the validation set (Nolen et al., 2011). Bigbee et al. (2012) evaluated a panel of 10 serum biomarkers comprising PRL, transthyretin, thrombospondin-1, E-selectin, C-C motif chemokine 5, macrophage migration inhibitory factor, plasminogen activator inhibitor, receptor tyrosine-protein kinase, CYFRA21, and serum amyloid A which together correctly classified lung cancer patients with 77.1% sensitivity and 76.2% specificity on the training set, and with 73.3% sensitivity and 93.3% specificity on the blinded verification set. Also, Yu et al. (2017) suggested circulating lipids can be developed as a potential biomarker for the lung cancer early detection and introduced a panel of four plasma lipid markers. Plasma lipid markers lysophosphatidylethanolamine (LPE(18:1)), egg phosphatidylethanolamine (ePE(40:4)), cholesteryl linoleate (C(18:2)CE), and sphingomyelin (SM(22:0)) distinguished early-staged NSCLC from healthy individuals with 81.9% sensitivity, 70.7% specificity and 82.3% accuracy on the training set and 78.7% sensitivity, 69.4% specificity and 80.8% accuracy on the validation set (Yu et al., 2017).

Most recently, Jiang et al. (2018) showed a tumor biomarker, thymidine kinase 1 (TK1) combined with CEA, CYFRA21, and neuron specific enolase (NSE) improves the diagnosis of the squamous cell carcinoma and adenocarcinoma subtypes. Mazzone et al. (2018) validated a panel of 3 serum biomarker proteins comprising CEA, carbohydrate antigen (CA125), CYFRA 21-1, and an auto-antibody New York esophageal cancer-1 (NY-ESO-1) together with clinical variables such as age, sex, including a clinical diagnosis of chronic obstructive pulmonary disease and smoking history were validated. They reported increase in AUC to 0.81 for the biomarker combined model with 96% specificity while sensitivity was low at 49%. Korkmaz et al. (2018) suggested a panel of three serum tumor biomarkers, CYFRA 21-1, HE4, and progastrin releasing peptide (ProGRP) that might contribute to discriminating lung cancer from benign lung cancer. They reported an increase in diagnostic value (AUC = 0.899) for CYFRA 21.1 combined with HE4 while ProGRP alone had the diagnostic value (AUC = 0.875) for discriminating SCLC from NSCLC (Korkmaz et al., 2018). Chu et al. (2018) reported the diagnostic performance of three biomarkers: an antibody based biomarker screening panel (Early CDT-lung), micro-RNA signature classifier (MSC) containing plasma-based 24 miRNA risk score and a serum-based 13 miRNA signature (miR-test). Clinical trials to study these biomarkers for lung cancer detection at early stage are currently ongoing (Chu et al., 2018).

## Conclusion

In conclusion, PRL could potentially be used as an effective serum biomarker for the diagnosis of NSCLC along with CEA and CYFRA21. Moreover, a combination of these three serum biomarkers seems more promising for the diagnosis of NSCLC than individual biomarkers alone. This study needs further validation with diverse population as the participants enrolled were mostly Chinese. Furthermore, comorbidities and co-existing conditions such as Tuberculosis, chronic obstructive pulmonary disease including smoking status of participants need to be considered. With more patients enrolled, the study could be extended to evaluate the performance of biomarkers in the subtypes of NSCLC.

## Availability of Data and Materials

The datasets used and/or analyzed during the current study are available from the corresponding authors on reasonable request.

## Author Contributions

RF managed the dataset, analyzed the data, interpreted the results, and drafted the manuscript. YZ designed the study and experiments, analyzed the data, and helped in manuscript drafting and revision. VK contributed significantly in literature search, and manuscript drafting and revision. BJ and FZ helped in literature search, and manuscript drafting and revision. YD designed the study and experiments, helped in analyzing the data, interpreting the results, and manuscript drafting and revision. All authors have read and approved the final manuscript.

## Conflict of Interest Statement

The authors declare that the research was conducted in the absence of any commercial or financial relationships that could be construed as a potential conflict of interest.
